# The Role of Total White Blood Cell Count in Antipsychotic Treatment for Patients with Schizophrenia

**DOI:** 10.2174/1570159X21666230104090046

**Published:** 2023-01-06

**Authors:** Yamin Zhang, Shiwan Tao, Jeremy Coid, Wei Wei, Qiang Wang, Weihua Yue, Hao Yan, Liwen Tan, Qi Chen, Guigang Yang, Tianlan Lu, Lifang Wang, Fuquan Zhang, Jianli Yang, Keqing Li, Luxian Lv, Qingrong Tan, Hongyan Zhang, Xin Ma, Fude Yang, Lingjiang Li, Chuanyue Wang, Liansheng Zhao, Wei Deng, Wanjun Guo, Xiaohong Ma, Dai Zhang, Tao Li

**Affiliations:** 1 Department of Neurobiology and Affiliated Mental Health Center, Hangzhou Seventh People's Hospital, Zhejiang University School of Medicine, Hangzhou, Zhejiang, China;; 2 Liangzhu Laboratory, MOE Frontier Science Center for Brain Science and Brain-Machine Integration, State Key Laboratory of Brain-Machine Intelligence, Zhejiang University, Hangzhou, Zhejiang, China;; 3 NHC and CAMS Key Laboratory of Medical Neurobiology, Zhejiang University, Hangzhou, China;; 4 Mental Health Center and Psychiatric Laboratory, West China Hospital of Sichuan University, Chengdu, Sichuan, China;; 5 Peking University Sixth Hospital (Institute of Mental Health), Beijing, China;; 6 National Clinical Research Center for Mental Disorders & Key Laboratory of Mental Health, Ministry of Health (Peking University), Beijing, China;; 7 Second Xiangya Hospital, Central South University, Changsha, Hunan, China;; 8 Beijing Anding Hospital, Beijing Institute for Brain Disorders, Capital Medical University, Beijing, China;; 9 Peking University HuiLongGuan Clinical Medical School, Beijing HuiLongGuan Hospital, Beijing, China;; 10 Wuxi Mental Health Center, Nanjing Medical University, Wuxi, Jiangshu, China;; 11 Institute of Mental Health, Tianjin Anding Hospital, Tianjin, China;; 12 Tianjin Medical University General Hospital, Tianjin Medical University, Tianjin, China;; 13 Hebei Mental Health Center, Baoding, Hebei, China;; 14 Second Affiliated Hospital of Xinxiang Medical University, Xinxiang, Henan, China;; 15 Department of Psychiatry, Xijing Hospital, Fourth Military Medical University, Xi'an, Shanxi, China

**Keywords:** Schizophrenia, antipsychotics, white blood cell, inflammation, drug response, metabolic alterations

## Abstract

**Background:**

Total white blood cell count (TWBCc), an index of chronic and low-grade inflammation, is associated with clinical symptoms and metabolic alterations in patients with schizophrenia. The effect of antipsychotics on TWBCc, predictive values of TWBCc for drug response, and role of metabolic alterations require further study.

**Methods:**

Patients with schizophrenia were randomized to monotherapy with risperidone, olanzapine, quetiapine, aripiprazole, ziprasidone, perphenazine or haloperidol in a 6-week pharmacological trial. We repeatedly measured clinical symptoms, TWBCc, and metabolic measures (body mass index, blood pressure, waist circumference, fasting blood lipids and glucose). We used mixed-effect linear regression models to test whether TWBCc can predict drug response. Mediation analysis to investigate metabolic alteration effects on drug response.

**Results:**

At baseline, TWBCc was higher among patients previously medicated. After treatment with risperidone, olanzapine, quetiapine, perphenazine, and haloperidol, TWBCc decreased significantly (*p* < 0.05). Lower baseline TWBCc predicted greater reductions in Positive and Negative Syndrome Scale (PANSS) total and negative scores over time (*p* < 0.05). We found significant mediation of TWBCc for effects of waist circumference, fasting low-density lipoprotein cholesterol, and glucose on reductions in PANSS total scores and PANSS negative subscale scores (*p* < 0.05).

**Conclusion:**

TWBCc is affected by certain antipsychotics among patients with schizophrenia, with decreases observed following short-term, but increases following long-term treatment. TWBCc is predictive of drug response, with lower TWBCc predicting better responses to antipsychotics. It also mediates the effects of certain metabolic measures on improvement of negative symptoms. This indicates that the metabolic state may affect clinical manifestations through inflammation.

**Clinical Trials Registration Number:**

Chinese Clinical Trials number: ChiCTR-TRC-10000934 (http://www.chictr.org.cn/showproj.aspx?proj=8604).

## 1 BACKGROUND

Schizophrenia is a severe mental disorder resulting in impaired daily functioning and working ability [1]. Although the underlying etiology remains unclear, several hypotheses have been proposed, including low-grade inflammation [2]. Because the involvement of inflammation in schizophrenia is now well recognized, it is important to focus on the identification of potential inflammatory indexes to aid assessment and management [3]. White blood cells (WBCs), composed of lymphocytes, monocytes, neutrophils, eosinophils, and basophils, produce almost all inflammatory mediators. TWBCc (total WBC count), including all these cells, is a readily available and inexpensive index. Previous studies have found that TWBCc was higher in patients with schizophrenia [4] and first-episode psychosis [5] than in healthy controls. Furthermore, consistent positive correlations between TWBCc and clinical symptoms assessed using the Positive and Negative Syndrome Scale (PANSS) and Brief Psychiatric Rating Scale (BPRS) have been found in patients with schizophrenia [6] and psychotic disorder [7].

TWBCc can be affected by antipsychotic medication. Neutropenia, diagnosed when the absolute neutrophil count becomes lower than 500/μL is a severe but uncommon side effect of some antipsychotics, such as clozapine and olanzapine [8]. Although neutropenia has gained wide attention, it is unknown how TWBCc changes within the normal range after taking antipsychotics. It is also of interest whether TWBCc change is associated with the efficacy of antipsychotics. We found a single study that followed 20 patients with persistent schizophrenia for 8 weeks: TWBCc was significantly decreased among patients whose negative symptoms improved, considered responders, following treatment with clozapine [9]. No significant change was found among non-responders, or responders with improved positive symptoms. This is a potentially important finding. However, the results need to be supported by further studies with larger sample sizes. Furthermore, it has not been studied whether different antipsychotics vary in their effects on TWBCc. In addition, the predictive value of TWBCc for efficacy of antipsychotics needs further study.

TWBCc is also associated with metabolic disturbances [10, 11] that are common in patients with schizophrenia, especially after treatment with antipsychotics [12]. Metabolic disturbances can lead to immune activation through different mechanisms, including alterations in integrity and shifts in TWBCc [13]. There is also evidence linking TWBCc to metabolic abnormalities in schizophrenia patients [12, 14, 15]. In a cross-sectional study, schizophrenia patients with metabolic syndrome had higher TWBCc [14]. In a longitudinal study, inflammatory markers predicted incident metabolic syndrome in patients with schizophrenia [15]. In general, it is believed that inflammation and metabolic dysfunction have synergistic effects that culminate in the development of metabolic problems [12]. In addition, metabolic disturbances are associated with antipsychotic response [16, 17]. As a result, metabolic disturbances may affect the antipsychotic response by changing TWBCc.

In this study, we first described longitudinal changes in TWBCc after monotherapy with different antipsychotics. To study the predictive value of TWBCc for antipsychotic efficacy, we compared TWBCc between patients with varied drug responses to antipsychotic treatment and then validated the results using a linear mixed model. This can accommodate unbalanced data patterns caused by dropout [18]. Finally, we explored whether metabolic disturbances can affect drug response through TWBCc using mediation analysis.

## 2 MATERIALS AND METHODS

### 2.1 Participants

This study, led by The Chinese Antipsychotics Pharmacogenomics Consortium, recruited 3,030 Han Chinese patients with schizophrenia from 32 different psychiatry departments across China between July 6, 2010, and November 30, 2011. All patients were aged from 18 to 45 and diagnosed by trained psychiatrists based on the criteria of schizophrenia using the Structured Clinical Interview for DSM-IV [19]. Exclusion criteria for all participants included 1) severe unstable physical diseases, such as immune disorder, diabetes, and thyroid diseases; 2) malignant syndrome and acute dystonia; 3) well-documented histories of epilepsy and hyperpyretic convulsion; 4) diagnosis of alcohol and drug dependence; 5) serious suicide attempt, or severe excitement and agitation; 6) abnormalities of the liver, renal or heart function; 7) pregnancy or breastfeeding; and 8) any reason resulting in the patient not being suitable to take drugs to which they might be assigned. In addition, no participants had taken medication for at least one week before the study. A detailed description of the protocol has been published elsewhere [20]. To minimize the potential influence of acute infection, we excluded patients who met any of the following criteria: 1) WBC identified in urine; and 2) recorded infection. We also excluded patients who developed neutropenia during the study according to side effect assessment.

The study was approved by the institutional review board at each site and was conducted following the Good Clinical Practice guidelines and the Helsinki Declaration. Written informed consent was provided to both the patients and their legal guardians. Chinese Clinical Trials number: ChiCTR-TRC-10000934 (http://www.chictr.org.cn/showproj.aspx?proj=8604).

### 2.2 Procedures

Participants were randomly assigned (1:1:1:1:1:1/2:1/2) to one of 5 atypical antipsychotics (risperidone, olanzapine, quetiapine, aripiprazole, and ziprasidone) and 2 typical antipsychotics (perphenazine and haloperidol) groups. Each patient took part in an interview with trained psychiatrists and completed a case report form (CRF) at baseline. Patients were then followed for 6 weeks and interviewed every two weeks. Demographic and clinical characteristics, including age, sex, age of onset, duration of illness (DOI), and psychiatric medication history, were collected at baseline. Anthropometric measures, including blood pressure, waist circumference, body height, and body weight, were assessed at baseline and every two weeks. DOI (months) was defined as the period between the onset of psychosis and the pretrial clinical interview. Waist circumference was taken at the end of normal expiration, measuring the minimum circumference at the level of the umbilicus to the nearest 0.5 cm. Body mass index (BMI) was calculated as weight divided by height squared (kg/m^2^).

### 2.3 Clinical Assessment

Psychiatrists assessed clinical symptoms using PANSS [21] every two weeks, namely at baseline, at the 2^nd^ week, 4^th^ week and 6^th^ week. PANSS reduction was calculated using the following formula:

PANSS reduction=100 × (PANSS score at baseline – PANSS score at the 6^th^ week) / (PANSS score at baseline – a).

In this formula, a is the sum when all items scored 1. For instance, a is 30 for PANSS total score, 7 for PANSS positive/negative subscale scores. Patients with PANSS reduction greater than 25% were defined as responders and the rest were non-responders.

### 2.4 Laboratory Assessments

Blood samples were collected between 8 and 9 am after an eight-hour fast. Blood laboratory analyses including TWBCc, glucose, total cholesterol, triglycerides, high-density lipoprotein cholesterol (HDL), and low-density lipoprotein cholesterol (LDL) were performed at the hospital where the blood samples were collected following a unified protocol at baseline and the 4^th^ week and 6^th^ week.

### 2.5 Statistical Analysis

We compared TWBCc at baseline between groups with different demographic and clinical characteristics using independent Student’s tests. The correlation between TWBCc and some continuous demographic and clinical variables was studied using Pearson correlation. We tested whether TWBCc changes over 6 weeks were significant in different antipsychotic groups using paired-samples T-tests. We compared TWBCc changes over time between responders and non-responders to antipsychotics using analysis of covariance (ANCOVA) adjusting for TWBCc at baseline and other variables associated with TWBCc, including sex, medication status, and DOI. To further evaluate TWBCc as a predictor for clinical improvement after antipsychotic treatment, mixed-effect linear regression models were fitted using the R package ‘lmerTest’. As fixed effects, we entered TWBCc at baseline, type and dosage of antipsychotics, and covariants that were associated with TWBCc. As random effects, we had intercept for subjects and slope for time (a 4-level factor). PANSS scores assessed during 4 different interviews were set as dependent variables. For mediation analyses, we first selected metabolic measures that were significantly associated with the reduction in PANSS total score, positive, or negative subscale scores (adjusted by FDR multiple testing). These measures were then considered predictors in the mediation model. The reduction in PANSS total score, positive or negative subscale score were outcomes in different models. TWBCc at baseline was set as the mediator, and the mediation analysis model was fitted using the R package ‘mediation’. Bootstrapping with 1000 random bootstrap samples was performed to obtain confidence intervals and *p* values. A schematic diagram of the mediation model is shown in Fig. (**S1**). For all analyses, extreme outlying data were winsorized at 1% or 99% for TWBCc and metabolic measures. The significance level was set at *p* < 0.05 (two-sided) or FDR q value < 0.05. All statistical analyses were performed with R software 4.1.0 (https://www.R-project.org/).

## 3 RESULTS

### 3.1 Characteristics of the Sample

**Fig. (1):**
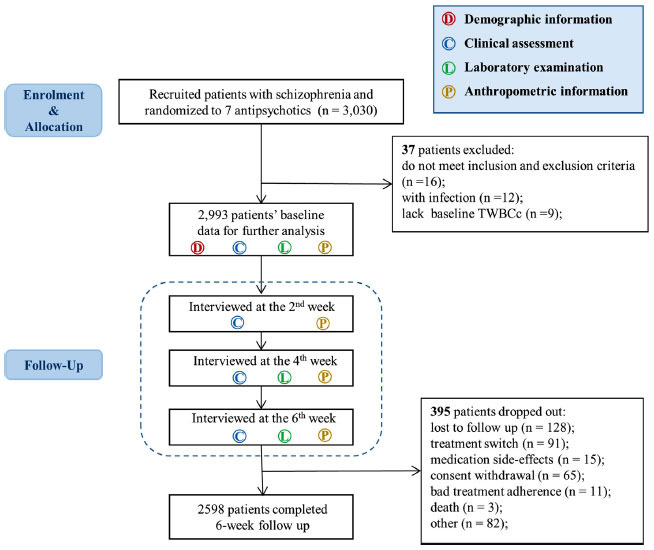
Trail profile.

A total of 3,030 patients with schizophrenia were recruited from the inpatient department of 32 hospitals. We further excluded 37 patients, including 16 patients by double checking the inclusion and exclusion criteria, 12 patients with infection and 9 patients without baseline TWBCc information. No patients developed neutropenia, resulting in 2993 patients included in the study. Mean age was 31.76 (SD 7.96); 1534 males (51.25%); and 865 were drug-naive (28.90%) (Fig. **1**). Details of the demographic, clinical, and laboratory characteristics of all participants at baseline are shown in Table **S1**. A total of 395 patients dropped out after the 6-week follow-up. These patients tended to be younger and drug naive. Comparisons between patients who dropped out and those who finished the study are shown in Table **S2**.

### 3.2 TWBCc at Baseline and Changes Over Time after Antipsychotic Treatment

**Fig. (2):**
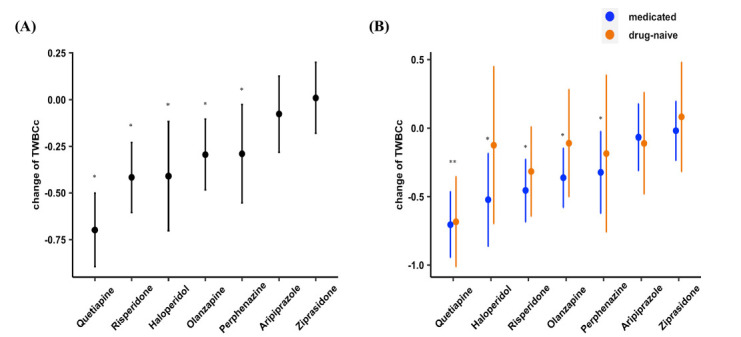
Longitudinal change of TWBCc after 6-week treatment with different antipsychotics. (**A**) Changes of TWBCc for patients in different antipsychotic groups; (**B**) Changes of TWBCc for drug-naive (orange) and medicated (blue) patients in different antipsychotic groups. Dots are the means of each group and lines represent the 95% confidence interval for those means.

**Fig. (3):**
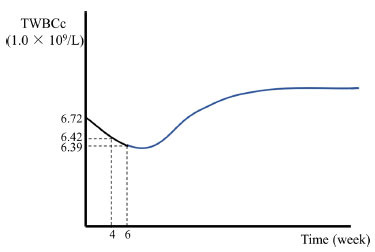
Bell-shaped change of TWBCc. The trajectory after 6 weeks (blue) was inferred based on the fact that patients with medication history before this trial had a higher level of TWBCc than those had never taken antipsychotics after adjusting for duration of illness.

Table **1** shows that male patients (compared to female patients) and medicated patients (compared to drug-naive patients) had higher TWBCc. TWBCc was also positively associated with DOI. To study whether the higher TWBCc in medicated patients was a result of medication or longer DOI, we adjusted for DOI and further compared medicated and unmedicated patients. Medication history was still associated with TWBCc (F = 5.18, *p* = 0.02) after adjustment. There was a significant decrease in TWBCc in almost all antipsychotic groups except for aripiprazole and ziprasidone (Fig. **2A** and Table **S3**). However, the decrease in drug-naive patients was minor and only significant among patients treated with quetiapine (Fig. **2B** and Table **S3**). Integrating these results, we infer that the trajectory of longitudinal change of TWBCc after antipsychotic treatment resembles a bell (Fig. **3**), namely a decrease after short-term treatment but an increase after long-term medication.

### 3.3 Predictive Value of TWBCc for Clinical Symptom Improvement

**Fig. (4):**
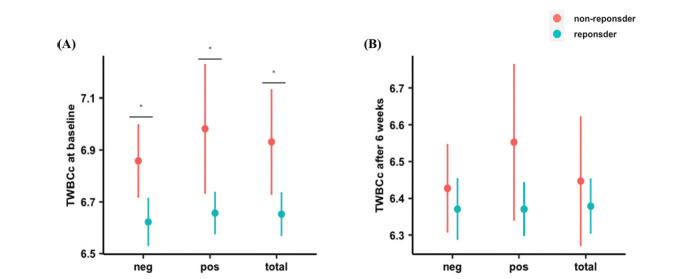
Average TWBCc in responders and non-responders. (**A**) TWBCc at baseline for responders (green) *vs*. non-responders (red); (**B**) TWBCc after 6-week treatment for responders *vs*. non-responders. Dots are the means of each group and lines represent the 95% confidence interval for those means.

In total, 2179 (72.8%), 2300 (76.8%), and 1805 (60.3%) patients achieved responses based on reductions in PANSS total scores, PANSS positive subscale scores, and PANSS negative subscale scores. We compared the difference in TWBCc between responders and non-responders and found that responders based on different PANSS scores all had lower TWBCc at baseline (*p* < 0.05) (Fig. **4A**). At the end of the study, differences in TWBCc were not significant between responders and non-responders (Fig. **4B**). We then checked the change of TWBCc over time and found that responders showed fewer changes in TWBCc than non-responders, despite adjusting for baseline TWBCc (Fig. **S2A**) or not adjusting (Fig. **S2B**). Mixed-effect linear regression models revealed that TWBCc at baseline could predict the change in PANSS total score and PANSS negative subscale score. For each 1.0 × 10^9^/L TWBCc decrease at baseline, PANSS total scores reduced by 0.31 (95% CI 0.06-0.56) and PANSS negative subscale scores reduced by 0.21 (95% CI 0.10-0.31) over 6 weeks.

### 3.4 Mediation Effect of TWBCc at Baseline

**Fig. (5):**
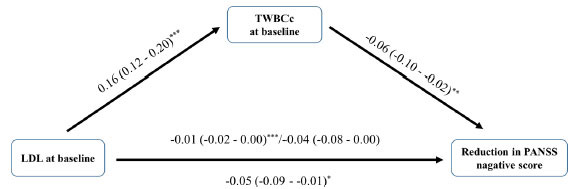
The mediation model of LDL affecting negative symptom improvement through TWBCc. *p < 0.05; **p < 0.01; ***p < 0.001. **Abbreviations:** LDL: low-density lipoprotein cholesterol; TWBCc: total white blood cell count; PANSS: positive and negative syndrome scale.

Three metabolic measures were associated with drug response after correction for multiple testing, including waist circumference, LDL, and glucose (Table **S4**). In 9 mediation models (3 metabolic measures × 3 kinds of reduction in PANSS scores), we found significant mediation of TWBCc at baseline for the effect of three metabolic measures (waist circumference, LDL, and glucose) on reduction in PANSS total scores, negative subscale scores, but not positive subscale scores (Table **S5**). The greatest mediation was identified for LDL, where 21.8% of the effect of LDL on the reduction in PANSS negative subscale scores was mediated by TWBCc at baseline (Fig. **5**).

## 4 DISCUSSION

In this large longitudinal cohort of patients with schizophrenia, we found that at baseline medicated patients have higher TWBCc than drug-naive patients, even after correcting for DOI. During follow-up, significant decreases in TWBCc were observed after treatment with certain antipsychotics, including risperidone, olanzapine, quetiapine, perphenazine, and haloperidol. TWBCc at baseline was predictive of drug response, where lower TWBCc at baseline predicted a greater reduction in PANSS total scores and PANSS negative subscale scores after treatment. We also found that TWBCc at baseline mediates the association between metabolic indices (waist circumference, LDL, and glucose at baseline) and drug response, defined by PANSS total scores and PANSS negative subscale scores.

### 4.1 The Longitudinal Change of TWBCc after Antipsychotic Treatment

We observed a significant decrease in TWBCc after antipsychotic treatment. This aligns with the inflammation hypotheses of schizophrenia which considers inflammation the source for developing the disease. This is supported by a large body of evidence about alterations in inflammation levels in unmedicated patients with first-episode schizophrenia. Our findings are in line with previous findings that antipsychotic treatment can resolve abnormally elevated blood cytokine levels, monocyte counts, neutrophil counts, and T-cell counts [12]. This can also explain why adjunctive anti-inflammatory drugs can improve clinical symptoms in the treatment of schizophrenia [22, 23].

The underlying mechanism through which antipsychotics reduce inflammation remains unexplained, but they are likely to affect multiple immune cells, both directly and indirectly. Firstly, antipsychotics can directly inhibit the activation of immune cells and production of pro-inflammatory cytokines by immune cells, including microglia activation [24, 25], Th1 polarization [26], cytokine production by activated microglia [24], peripheral blood monocytes [27], *etc*. This antipsychotic-induced reduction in cytokines can indirectly suppress monocyte activation [28] and neutrophil chemotaxis [29]. In addition, because stress can cause neuroinflammation [30], antipsychotics may limit stress-induced neuroinflammation by diminishing the stress of patient experiences during psychotic episodes. It is thought that mild but common reduction of TWBCc after antipsychotic treatment is totally different from neutropenia, which is a rare and fatal side effect of some antipsychotics. Recent theory explaining neutropenia includes an immune-mediated response against haptenized neutrophils. In contrast to suppressing monocyte activation, the proliferative response of peripheral blood monocytes was activated after neutrophils were haptenized by antipsychotics [31].

However, the decrease of TWBCc following antipsychotic treatment conflicts with results showing medicated patients have higher TWBCc than drug-naive patients even after correcting for DOI. Similar phenomena have been reported for pro-inflammatory cytokines such as IL-1β and IL-6 whose longitudinal change resembles a bell-shaped curve. Multiple studies have reported that levels of IL-6 and IL-1β are decreased after treatment with typical or atypical antipsychotics. However, in the long term, their levels either rise or do not change compared to baseline levels [32]. This might result from the various degrees of side effects of weight gain and other metabolic alterations which are closely associated with elevated production of chronic inflammation. We have also reported significant increases in body weight, blood lipids, and glucose [33]. Because TWBCc changes are bell-shaped, this might reflect the net effect of the direct anti-inflammatory impact of antipsychotics and elevated inflammation levels associated with weight gain and other metabolic alterations over a longer period. In addition, the bell-shaped change of inflammation markers may explain why schizophrenia has a long treatment period and high relapse rate, even though a short-term response is achieved.

### 4.2 The Predictive value of TWBCc for Clinical Symptom Improvement

A preliminary comparison between responders and non-responders found that non-responders had higher TWBCc at baseline, followed by a greater decrease, and ended the study with no significant differences. The greater decrease among non-responders was mainly driven by a higher baseline value, because more differences were observed when TWBCc at baseline was unadjusted. A similar phenomenon was observed for metabolic measures in a previous study where more severe metabolic deterioration was observed among patients with better metabolic conditions at baseline. For example, patients with lower BMI gained more weight [33]. It seems that patients tend to converge after antipsychotic treatment. However, this is not consistent with a previous study where TWBCc decreased among responders but increased slightly among non-responders after treatment with clozapine [9]. The previous study included patients with persistent schizophrenia, who we excluded from this study and had a smaller sample size, with less than 10 responders. It also followed the patients for 2 weeks longer than this study. We cannot tell which of these methodological differences explained the different results. However, we would suggest validating the phenomenon among patients with persistent schizophrenia in a larger sample before further investigating the mechanism.

Our linear mixed model supported the predictive value of TWBCc at baseline for overall improvement and negative symptom improvement, but not positive symptom improvement. A previous study has also reported that increased levels of inflammatory markers, in particular IL-6 and IFN-γ, were associated with poor treatment response, and IFN-γ was more strongly associated with the severity of negative symptoms [34]. In addition, associations between the improvement of clinical symptoms and metabolic alterations, such as weight gain [35] and increased blood lipid [36], have been validated. In this study, we also observed a positive association between TWBCc and metabolic measures at baseline, more weight gain in patients with lower BMI at baseline, and more blood lipid increase in patients with lower blood lipid levels [37]. Integrating all these findings, we can see that lower TWBCc means lower BMI and blood lipid levels, more weight gain and blood lipid increase after treatment, and thus better drug response. Nevertheless, TWBCc is less associated with positive symptom improvement, while associations of metabolic alterations do not have this bias. The reason for this might be that inflammation is more involved in negative symptoms than positive symptoms. This is supported by the fact that more cytokines have been found to be associated with negative symptoms than positive symptoms [38] and more improvement in negative symptoms than positive symptoms after adding anti-inflammatory treatment to antipsychotics [39].

The mechanisms underlying these associations are unclear. We suggest potential mechanisms based on the literature review, but future experimental studies are needed to investigate this question. It is recognized that neuroinflammation can do extensive harm to neurons, including impairing neurogenesis, facilitating neurodegenerative processes, *etc*. [40]. As a result, patients with a high level of inflammation may have a poor response to antipsychotics due to severer neurological damage. In addition, the anti-inflammatory effect of antipsychotics is limited, so patients with higher inflammation levels may need add-on anti-inflammatory treatment to achieve a better response. However, according to the latest meta-analysis, no study on the efficacy of anti-inflammatory agents for patients with schizophrenia has stratified patients according to the presence of immune alterations [41]. Apart from influencing neurons, inflammation can also disrupt blood-brain barrier endothelial function *via* the secretion of reactive oxygen species and cytokines [42]. Given any antipsychotics must cross the blood-brain barrier to act on the brain, drug response may be affected by blood-brain barrier function.

### 4.3 Mediation Effect of TWBCc at Baseline

We found significant mediation of TWBCc in the association between three metabolic measures (waist circumference, LDL, and glucose) and reduction in PANSS total sores, negative subscale scores, but not positive subscale scores. Although the underlying mechanism is unknown, there are reports on the potential pathogenesis of the mediation of inflammation in the progression from metabolic alterations to metabolic diseases. It is reported that adipocyte metabolic alterations lead to the accumulation and activation of leukocytes in adipose tissues, ultimately resulting in metabolic diseases such as insulin resistance [43-46]. The fact that leukocyte changes occurred mainly in adipose tissue corresponds to a larger mediation proportion for LDL than other metabolic measures. While the importance of managing physical health in patients with chronic schizophrenia to control cardiovascular diseases risk has gained worldwide attention, the path from improvement in metabolic condition to reduce inflammation and then to ameliorate symptoms has drawn less attention [47].

### 4.4 Strengths and Limitations

The strengths of this study are that we assessed patients using PANSS in a longitudinal cohort and studied the association between PANSS scores and TWBCc in a larger sample of patients than in other studies. In addition, we also measured several metabolic indices, which enabled us to study the relationship among measures reflecting three different aspects of patients. There are certain limitations to this study. First, only the total WBC count but no subtype WBC counts were recorded in CRF designed for the clinical trial. However, the subtype WBC count may offer more information. For example, monocyte count can be considered an indirect marker of microglial activation in the central nervous system [48]. Second, we only followed the patients for 6 weeks, which is not long enough to observe the potential bell-shaped change of TWBCc.

## CONCLUSION AND FUTURE PROSPECTS

The longitudinal change of TWBCc following antipsychotic treatment resembled a bell shape. We also found that lower TWBCc at baseline can predict a better response to antipsychotics, especially negative symptoms. This indicates adjunctive treatment other than antipsychotics can further improve negative symptoms for patients with high TWBCc at baseline. Further studies are needed to test this hypothesis. Higher levels of TWBCc are associated with a worse metabolic condition at baseline. It also partly mediates the negative association between metabolic measures and negative symptom improvement. This indicates that improvement in the management of metabolic measures will benefit decreasing TWBCc and thus leads to better drug response.

## Figures and Tables

**Table 1:** Demographic, clinical and laboratory characteristics of all participants.

**Variables**	**TWBCc** ^Π^ **Mean (SD)**	***t***	***p* Value**
**Sex**	-	4.22	<0.001
Men	6.87 (2.00)	-	-
Women	6.56 (1.95)	-	-
**Medication**	-	2.34	0.02
Drug-naive	6.59 (1.86)	-	-
Medicated	6.77 (2.03)	-	-
-	**r**	**95% CI**	***p* Value**
**Age**	0.03	-0.01 - 0.07	0.09
**Educational years**	-0.03	-0.06 - 0.01	0.16
**Age of onset**	-0.00	-0.12 - 0.03	0.91
**DOI (month)**	0.05	0.01 - 0.08	0.009

**Note**: Π : ×10^9^/L; DOI: duration of illness; TWBCc: total white blood cell count.

## Data Availability

Available in electronic form upon reasonable request.

## LIST OF ABBREVIATIONS

BMI: Body Mass Index
BPRS: Brief Psychiatric Rating Scale
CRF: Case Report Form
DOI: Duration of Illness
HDL: High-density Lipoprotein Cholester
LDL: Low-density Lipoprotein Cholesterol
PANSS: Positive and Negative Syndrome Scale
TWBCc: Total White Blood Cell Count
WBCs: White Blood Cells
