# Supplementary material for: The Role of Total White Blood Cell Count in Antipsychotic Treatment for Patients with Schizophrenia
Source: Curr Neuropharmacol. 2023 Jan 6;22(1):159–67. doi: 10.2174/1570159X21666230104090046 (PMC10716888; doi:10.2174/1570159X21666230104090046)
Supplement: Supplementary file 1 [file CN-22-159_SD1.pdf]

Supplementary Material

The Role of Total White Blood Cell Count in Antipsychotic Treatment for Patients with Schizophrenia

Yamin Zhang[1,2,3,#], Shiwan Tao[4,#], Jeremy Coid[4], Wei Wei[1,2,3], Qiang Wang[4], Weihua Yue[5,6], Hao Yan[5,6], Liwen Tan[7], Qi Chen[8], Guigang Yang[9], Tianlan Lu[5,6], Lifang Wang[5,6], Fuquan Zhang[10], Jianli Yang[11,12], Keqing Li[13], Luxian Lv[14], Qingrong Tan[15], Hongyan Zhang[5,6], Xin Ma[8], Fude Yang[9], Lingjiang Li[7], Chuanyue Wang[8], Liansheng Zhao[4], Wei Deng[1,2,3], Wanjun Guo[1,2,3], Xiaohong Ma[4], Dai Zhang[5,6] and Tao Li[1,2,3,*]

[1]Department of Neurobiology and Affiliated Mental Health Center, Hangzhou Seventh People's Hospital, Zhejiang University School of Medicine, Hangzhou, Zhejiang, China; [2]Liangzhu Laboratory, MOE Frontier Science Center for Brain Science and Brain-Machine Integration, State Key Laboratory of Brain-Machine Intelligence, Zhejiang University, Hangzhou, Zhejiang, China; [3]NHC and CAMS Key Laboratory of Medical Neurobiology, Zhejiang University, Hangzhou, China; [4]Mental Health Center and Psychiatric Laboratory, West China Hospital of Sichuan University, Chengdu, Sichuan, China; [5]Peking University Sixth Hospital (Institute of Mental Health), Beijing, China; [6]National Clinical Research Center for Mental Disorders & Key Laboratory of Mental Health, Ministry of Health (Peking University), Beijing, China; [7]Second Xiangya Hospital, Central South University, Changsha, Hunan, China; [8]Beijing Anding Hospital, Beijing Institute for Brain Disorders, Capital Medical University, Beijing, China; [9]Beijing HuiLongGuan Hospital, Beijing, China; [10]Wuxi Mental Health Center, Nanjing Medical University, Wuxi, Jiangshu, China; [11]Institute of Mental Health, Tianjin Anding Hospital, Tianjin, China; [12]Tianjin Medical University General Hospital, Tianjin Medical University, Tianjin, China; [13]Hebei Mental Health Center, Baoding, Hebei, China; [14]Second Affiliated Hospital of Xinxiang Medical University, Xinxiang, Henan, China; [15]Department of Psychiatry, Xijing Hospital, Fourth Military Medical University, Xi'an, Shanxi, China

1875-6190/24 $65.00+.00
© 2024 Bentham Science Publishers

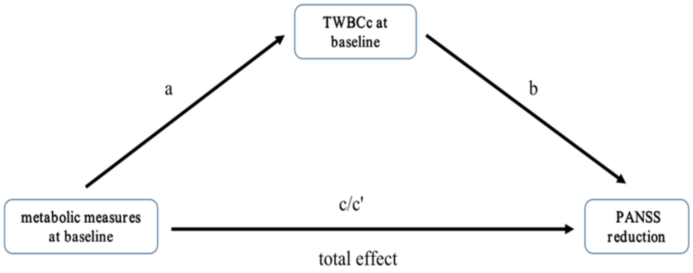

Fig. (S1). Schematic diagram of the mediation model. (**a**) estimate of effect of metabolic measures on TWBCc at baseline; (**b**) estimate effect of TWBCc at baseline on PANSS reduction; (**c**) indirect effect, c': direct effect.

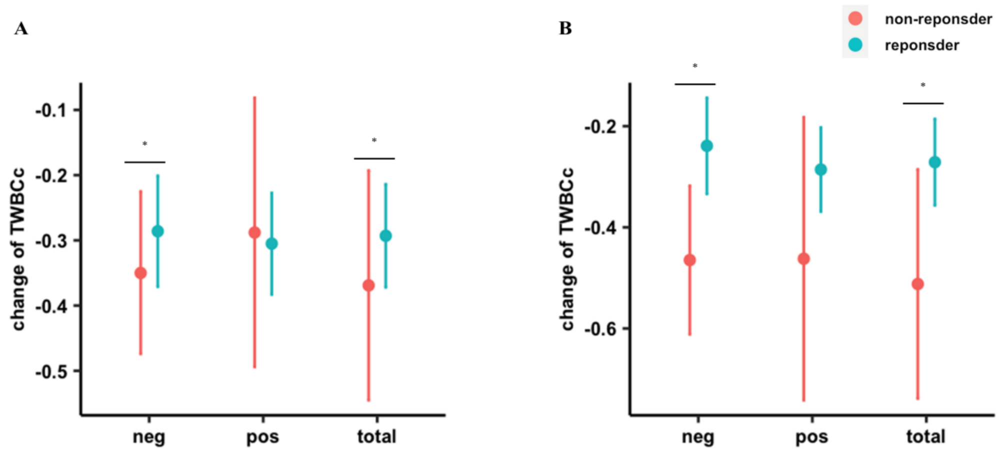

Fig. (S2). Change of TWBCc in responders and non-responders. (**A**) changes of TWBCc for responders *vs*. non-responders after adjustment for TWBCc at baseline and other covariants; groups defined according to scores of PANSS negative subscale (F = 6.96, p = 0.008), to scores of overall total items (F = 10.46, p = 0.001), and to scores of PANSS positive subscale (F = 2.67, p = 0.10); (**B**) changes of TWBCc for responders *vs*. non-responders without any adjustment. TWBCc: total white blood cell count; *p < 0.05.

Table S1. Demographic, clinical and laboratory characteristics of drug-naïve and medicated patients.

Variable	Total	Drug-naïve	Medicated	t/χ^2	*p* value
	(n=2993)	(n=865)	(n=2128)		
TWBCc[п]	6.72±1.98	6.59±1.86	6.77±2.03	2.34	0.019
Age	31.76±7.96	29.8±7.73	32.85±7.79	12.08	<0.001
Male	1534 (51.25%)	449 (51.91%)	367 (19.36%)	0.17	0.677
Edu years	10.40±3.28	10.43±3.37	10.39±3.25	-0.32	0.748
Family history	639 (21.44%)	174 (20.19%)	465 (21.94%)	1.02	0.312
Age of onset	25.29±6.99	26.85±7.49	24.66±6.67	-7.48	<0.001
DOI (years)	6.17±5.94	2.19±3.48	7.78±5.97	31.78	<0.001
**PANSS subscale**					
Total	89.46±15.32	88.65±15.49	89.79±15.23	1.83	0.060
Positive	25.52±4.71	25.54±4.68	25.51±4.72	-0.16	0.873
Negative	21.77±6.74	21.01±6.91	22.08±6.64	3.88	<0.001
General	42.15±8.43	42.09±8.40	42.17±8.45	0.24	0.812
**Metabolic measures**					
BMI[a]	22.23±3.70	21.33±3.35	22.59±3.78	9.01	<0.001
Waist circumference[b]	79.60±11.37	76.95±10.75	80.68±11.45	8.44	<0.001
Systolic blood pressure	115.98±11.60	115.52±11.84	116.17±11.50	1.38	0.166
Diastolic blood pressure	75.66±8.38	75.25±8.32	75.82±8.40	1.67	0.093
Glucose[c]	4.85±0.77	4.74±0.70	4.89±0.79	5.21	<0.001
Total cholesterol[c]	4.18±0.92	4.13±0.92	4.21±0.92	2.22	0.026
Triglycerides[c]	1.23±0.72	1.16±0.66	1.26±0.74	3.88	<0.001
HDL[c]	1.32±0.36	1.34±0.35	1.30±0.36	-2.71	0.006
LDL[c]	2.36±0.86	2.26±0.80	2.40±0.88	4.07	<0.001

Mean (SD) and n (%) were presented for continuous and categorical variables respectively.
BMI: body mass index; DOI: duration of illness; HDL: high-density lipoprotein cholesterol; LDL: low-density lipoprotein cholesterol; WBC: white blood cell.
[п]: $\times 10^9$/L; [a] kg/m^2; [b]cm; [c] mmol/L

Table S2. Characteristics of patients who dropped out.

Variables	All	Dropout before 2w n=138	Dropout after 2w and before 4 w n=113	Dropout after 4w n=138	χ^2/F	*p* value	Pairwise Comparison
Sex (male)	1530 (51.3)	69 (50.0)	56 (49.6)	70 (50.7)	1.71	0.19	
Drug naive	863 (28.9)	62 (44.9)	37 (32.7)	55 (39.9)	532.2	<0.001	
Family history	637 (21.4)	29 (21.0)	28 (24.8)	24 (17.4)	1103.4	<0.001	
Age	31.77±7.96	29.89±8.13	30.64±8.28	29.33±7.98	6.63	<0.001	all>after 4w, all > before 2w
Educational years	10.41±3.29	11.32±3.34	10.79±3.62	10.46±3.16	3.76	0.01	all < before 2w
Age of onset	25.30±6.99	24.35±6.56	25.25±6.57	24.17±6.96	1.89	0.12	
DOI (years)	6.17±5.94	5.32±6.12	4.81±5.43	4.67±5.67	5.21	0.001	all > after 4w

Mean (SD) and n (%) were presented for continuous and categorical variables respectively.
DOI: duration of illness.

Table S3. TWBCc change in different antipsychotic groups.

Antipsychotics	Mean±SD	Total (n=2993)	Drug-naïve (n=865)	Medicated (n=2 128)
		t/p[a]	*t/p*[a]	*t/p*[a]
Aripiprazole	-0.08±2.12	0.74/0.459	0.58/0.563	0.53/0.595
Haloperidol	-0.41±2.02	2.75/0.007	0.42/0.675	3.02/0.003
Olanzapine	-0.29±1.99	3.05/0.002	0.54/0.589	3.29/0.001
Perphenazine	-0.29±1.88	2.15/0.033	0.63/0.531	2.12/0.035
Quetiapine	-0.7±2.02	6.94/<0.001	4.06/<0.001	5.76/<0.001
Risperidone	-0.42±1.93	4.35/<0.001	1.90/0.06	3.91/<0.001
Ziprasidone	0.01±1.93	-0.09/0.926	-0.41/0.685	0.17/0.863

[a]Dependent Student's test for each treatment group.

Table S4. Association between reduction in PANSS scores and metabolic measures.

Drug Response	Metabolic	r	95% CI_L	95% CI_U	*p value*	FDR q value
reduction_total	BMI	-0.03	-0.07	0.00	0.085	0.198
reduction_total	waist circumference	-0.09	-0.13	-0.05	<0.001	**<0.001**
reduction_total	Total cholesterol	0.00	-0.04	0.03	0.801	0.885
reduction_total	Triglycerides	-0.03	-0.07	0.01	0.120	0.210
reduction_total	LDL	-0.06	-0.10	-0.02	0.006	**0.020**
reduction_total	HDL	-0.01	-0.05	0.03	0.662	0.817
reduction_total	Glucose	-0.06	-0.09	-0.02	0.005	**0.020**
reduction_pos	BMI	-0.02	-0.06	0.02	0.301	0.451
reduction_pos	Waist circumference	-0.07	-0.11	-0.03	<0.001	**0.002**
reduction_pos	Total cholesterol	0.01	-0.03	0.05	0.558	0.781
reduction_pos	Triglycerides	0.00	-0.04	0.04	0.995	0.995
reduction_pos	LDL	-0.03	-0.07	0.01	0.137	0.222
reduction_pos	HDL	-0.01	-0.05	0.03	0.647	0.817
reduction_pos	Glucose	-0.03	-0.07	0.01	0.105	0.210
reduction_neg	BMI	-0.03	-0.07	0.01	0.116	0.210
reduction_neg	Waist circumference	-0.10	-0.14	-0.06	<0.001	**<0.001**
reduction_neg	Total cholesterol	0.00	-0.04	0.04	0.901	0.946
reduction_neg	Triglycerides	-0.04	-0.08	-0.01	0.023	0.061
reduction_neg	LDL	-0.05	-0.09	-0.01	0.018	0.055
reduction_neg	HDL	-0.01	-0.04	0.03	0.792	0.885
reduction_neg	Glucose	-0.07	-0.10	-0.03	0.001	**0.005**

r: coefficient of Pearson's correlation; 95%CI: 95% confidence interval;
BMI: body mass index; HDL: high-density lipoprotein cholesterol; LDL: low-density lipoprotein cholesterol.

Table S5. The mediation effect of three metabolic measures between baseline TWBCc and reduction in PANSS sores.

Metabolic Measures	Drug Response	a β (95% CI)	b β (95% CI)	c β (95% CI)	c' β (95% CI)	Total Effect β (95% CI)	% Mediated
Waist circumference	reduction_total	0.14 (0.10-0.18)***	-0.05 (-0.09-0.01)*	-0.01 (-0.01-0.00)*	-0.08 (-0.12-0.05)***	-0.09 (-0.13-0.05)***	7.3%
Waist circumference	reduction_pos	0.14 (0.11-0.18)***	-0.02 (-0.06-0.01)	0.00 (-0.01-0.00)	-0.07 (-0.11-0.03)***	-0.07 (-0.11-0.03)***	4.6%
Waist circumference	reduction_neg	0.14 (0.11-0.18)***	-0.06 (-0.10-0.02)**	-0.01 (-0.01-0.00)**	-0.09 (-0.13-0.05)***	-0.10 (-0.13-0.06)***	8.4%
LDL	reduction_total	0.16 (0.12-0.20)***	-0.05 (-0.09-0.01)*	-0.01 (-0.02-0.00)**	-0.05 (-0.09-0.01)*	-0.06 (-0.09-0.02)***	14.9%
LDL	reduction_pos	0.16 (0.12-0.20)***	-0.03 (-0.07-0.01)	-0.01 (-0.01-0.00)	-0.03 (-0.06-0.01)	-0.03 (-0.07-0.01)	15.0%
LDL	reduction_neg	0.16 (0.12-0.20)***	-0.06 (-0.10-0.02)**	-0.01 (-0.02-0.00)***	-0.04 (-0.08-0.00)	-0.05 (-0.09-0.01)*	21.8%
Glucose	reduction_total	0.14 (0.11-0.18)***	-0.05 (-0.09 -0.01)**	-0.01 (-0.01-0.00)**	-0.05 (-0.09-0.01)*	-0.05 (-0.09-0.01)**	13.8%
Glucose	reduction_pos	0.14 (0.11-0.18)***	-0.03 (-0.07-0.01)	0.00 (-0.01-0.00)	-0.03 (-0.07-0.01)	-0.03 (-0.07-0.00)	12.1%
Glucose	reduction_neg	0.14 (0.11-0.18)***	-0.06 (-0.10-0.02)**	-0.01 (-0.02-0.00)***	-0.06 (-0.09-0.02)**	-0.07 (-0.10-0.03)**	13.7%

a: estimate of effect of metabolic measures on TWBCc at baseline; b: estimate effect of TWBCc at baseline on PANSS reduction ; c: indirect effect; c': direct effect; LDL: low-density lipoprotein cholesterol; neg: PANSS negative subscale score; pos: PANSS positive subscale score; total: PANSS total score.

*$p < 0.05$; **$p < 0.01$; ***$p < 0.001$.
